# Four-and-a-half LIM domains proteins are novel regulators of the protein kinase D pathway in cardiac myocytes

**DOI:** 10.1042/BJ20131026

**Published:** 2014-01-10

**Authors:** Konstantina Stathopoulou, Friederike Cuello, Alexandra J. Candasamy, Elizabeth M. Kemp, Elisabeth Ehler, Robert S. Haworth, Metin Avkiran

**Affiliations:** *Cardiovascular Division, King's College London British Heart Foundation Centre, London SE1 7EH, U.K.; †Institute of Experimental Pharmacology and Toxicology, University Medical Center Hamburg-Eppendorf, Martinistr. 52, Hamburg 20146, Germany

**Keywords:** cardiac myocyte, four-and-a-half LIM (FHL), histone deacetylase, neurohormonal stimulation, protein kinase, signal transduction, ARVM, adult rat ventricular myocyte, BPKDi, bipyridyl PKD inhibitor, CaMK, Ca^2+^/calmodulin-dependent protein kinase, caPKD, constitutively active catalytic domain of PKD, cMyBP-C, cardiac myosin-binding protein C, CRM1, chromosome region maintenance 1, cTnI, inhibitory subunit of cardiac troponin, ERK, extracellular-signal-regulated kinase, ET1, endothelin 1, FHL, four-and-a-half LIM domains, HDAC, histone deacetylase, IVK, *in vitro* kinase, MEF2, myocyte enhancer factor 2, MOI, multiplicity of infection, MuRF, muscle RING finger, NRVM, neonatal rat ventricular myocyte, PE, phenylephrine, pfu, plaque-forming unit, PKC, protein kinase C, PKD, protein kinase D, TAC, transverse aortic constriction

## Abstract

PKD (protein kinase D) is a serine/threonine kinase implicated in multiple cardiac roles, including the phosphorylation of the class II HDAC5 (histone deacetylase isoform 5) and thereby de-repression of MEF2 (myocyte enhancer factor 2) transcription factor activity. In the present study we identify FHL1 (four-and-a-half LIM domains protein 1) and FHL2 as novel binding partners for PKD in cardiac myocytes. This was confirmed by pull-down assays using recombinant GST-fused proteins and heterologously or endogenously expressed PKD in adult rat ventricular myocytes or NRVMs (neonatal rat ventricular myocytes) respectively, and by co-immunoprecipitation of FHL1 and FHL2 with GFP–PKD1 fusion protein expressed in NRVMs. *In vitro* kinase assays showed that neither FHL1 nor FHL2 is a PKD1 substrate. Selective knockdown of FHL1 expression in NRVMs significantly inhibited PKD activation and HDAC5 phosphorylation in response to endothelin 1, but not to the α_1_-adrenoceptor agonist phenylephrine. In contrast, selective knockdown of FHL2 expression caused a significant reduction in PKD activation and HDAC5 phosphorylation in response to both stimuli. Interestingly, neither intervention affected MEF2 activation by endothelin 1 or phenylephrine. We conclude that FHL1 and FHL2 are novel cardiac PKD partners, which differentially facilitate PKD activation and HDAC5 phosphorylation by distinct neurohormonal stimuli, but are unlikely to regulate MEF2-driven transcriptional reprogramming.

## Abstract 2

Protein kinase D has multiple roles in cardiac myocytes, where its regulatory mechanisms remain incompletely defined. In the present study we identify four-and-a-half LIM domains proteins 1 and 2 as novel binding partners and regulators of protein kinase D in this cell type.

## INTRODUCTION

The PKD (protein kinase D) family of serine/threonine kinases consists of three members, PKD1, PKD2 and PKD3, and belongs to the CaMK (Ca^2+^/calmodulin-dependent protein kinase) superfamily. These PKD isoforms share the common structural features of a C-terminal catalytic domain and an N-terminal regulatory domain. Components of the regulatory domain autoinhibit the activity of the catalytic domain in unstimulated cells and promote PKD association with the plasma and intracellular membranes after stimulation with hormones, growth factors, neurotransmitters, chemokines and bioactive lipids [1,2].

In cardiac myocytes, the most abundantly expressed PKD family member is PKD1, which is activated after stimulation of diverse GPCRs (G-protein-coupled receptors) that signal via Gα_q_, including α_1_-adrenergic, ET1 (endothelin 1) and angiotensin II receptors [3–5]. The principal PKD activation mechanism involves recruitment of the kinase to plasma or intracellular membranes by DAG (diacylglycerol) and transphosphorylation of its activation loop at amino acid residues Ser^744^ and Ser^748^ (amino acid numbering refers to murine PKD1) by activated novel PKC (protein kinase C) isoforms. The resulting PKD activation then leads to both autophosphorylation at residue Ser^916^ and transphosphorylation of PKD substrates, which include transcription factors, proteins involved in cell motility and vesicle fission from the Golgi apparatus, other kinases and sarcomeric proteins [1,2,6].

The functional significance of PKD1 in cardiac myocyte (patho)physiology has recently started to be unveiled by both *in vitro* and *in vivo* studies. We have shown previously that PKD1 may regulate cardiac myofilament function and the Ca^2+^ sensitivity of contraction by phosphorylating cTnI (inhibitory subunit of cardiac troponin) at Ser^22^/Ser^23^ [7,8] and cMyBP-C (cardiac myosin-binding protein C) at Ser^302^ [9]. Furthermore, PKD1 has been proposed to facilitate cardiac hypertrophy through the phosphorylation of HDAC5 (histone deacetylase isoform 5) at Ser^259^ and Ser^498^ [10]. Nuclear HDAC5 associates with and represses the activity of MEF2 (myocyte enhancer factor 2) transcription factors, which drive the transcriptional reprogramming that precipitates pathological cardiac hypertrophy and remodelling. In response to pro-hypertrophic neurohormonal stimuli, activated PKD1 phosphorylates HDAC5 at Ser^259^ and Ser^498^, thus inducing the binding of 14-3-3 proteins to these sites and revealing a NES (nuclear export sequence) that triggers HDAC5 extrusion from the nucleus to the cytosol, through a mechanism that is mediated by the CRM1 (chromosome region maintenance 1) protein [10,11]. HDAC5 nuclear export de-represses MEF2 transcriptional activity, which then drives pro-hypertrophic gene expression [12–14]. Studies in mice with cardiac-specific deletion [15] or overexpression [16] of PKD1 corroborate a key role for PKD1 in pathological cardiac remodelling, and PKD1 expression and activation have been shown to be increased in failing human myocardium [17].

The key roles proposed for PKD activity in cardiac (patho)physiology make improved understanding of the molecular mechanisms underlying both the upstream regulation and the downstream actions of this kinase in the heart an imperative. Towards this objective, in a previous study [7], we performed a yeast two-hybrid screen of a human cardiac cDNA library, which identified FHL2 (four-and-a-half LIM domains protein 2) as a novel binding partner for the PKD1 catalytic domain. In the present study, we have confirmed and characterized the interaction of full-length PKD1 with FHL2 as well as the highly homologous FHL isoform FHL1 (both of which are abundantly expressed in the heart [18]) in cardiac myocytes and explored the potential functional significance of these FHL isoforms in regulating PKD activity and downstream actions in that cell type.

## EXPERIMENTAL

### Materials

Rabbit polyclonal antibodies against phosphorylated (pSer^744^/Ser^748^ and pSer^916^) PKD were from Cell Signaling Technology. Rabbit polyclonal antibodies against total PKD were from Cell Signaling Technology and Santa Cruz Biotechnology. A rabbit polyclonal antibody against phosphorylated (pSer^498^) HDAC5 and a mouse monoclonal antibody against FHL1 were from Abcam. A mouse monoclonal antibody against FHL2 was from Medical and Biological Laboratories. Mouse monoclonal antibodies against GFP and GST were from Roche and Santa Cruz Biotechnology respectively. Expression vectors for expression of recombinant mouse FHL1 and human FHL2 proteins were gifts from Stephan Lange (Department of Medicine, UC San Diego, CA, U.S.A.) [19]. Recombinant PKD catalytic domain expressed in Sf21 insect cells was a gift from Harold Jeffries and Peter J. Parker (London Research Institute, Cancer Research UK) and recombinant human TnI was a gift from Douglas G. Ward and Ian Trayer (School of Biosciences, University of Birmingham, Birmingham, U.K.) [7]. BPKDi (bipyridyl PKD inhibitor) was custom-synthesized by the MRC Protein Phosphorylation Unit, University of Dundee, Dundee, U.K. [20]. The adenoviral vectors used to express mouse PKD1 (AdV:wtPKD1) and enhanced GFP (AdV:GFP) were prepared as described previously [8]. The adenoviral vector encoding human PKD1 tagged at the N-terminus with GFP (AdV:GFP–PKD1) was a gift from Jody Martin (Department of Cell and Molecular Physiology, Loyola University Chicago, IL, U.S.A.) [17]. The MEF2-luciferase reporter construct was a gift from Eric Olson (Department of Molecular Biology, UT Southwestern, TX, U.S.A.) [14] and the promoter from this (comprising three tandem repeats of the MEF2-binding site from the desmin enhancer) was subcloned into a pGL4.24 vector encoding the synthetic luciferase reporter gene *luc2P* (Promega). The vector encoding *Renilla* luciferase reporter (pRL-null) was from Promega. ET1 was from Calbiochem. PMA and PE (phenylephrine) were from Sigma–Aldrich. Secondary antibodies and ECL kits were from GE Healthcare. All other chemicals were from Sigma–Aldrich, Life Technologies or Fisher Scientific, unless otherwise stated. The investigation was performed in accordance with the Home Office Guidance on the Operation of the Animals (Scientific Procedures) Act 1986 (U.K.).

### Isolation and culture of ARVMs (adult rat ventricular myocytes)

ARVMs were isolated and maintained in culture as described previously [8]. Attached cardiac myocytes were transduced with an adenoviral vector expressing murine PKD1 at a MOI (multiplicity of infection) of 100 pfu (plaque-forming unit)/cell. ARVMs were maintained in culture for 24 h before use in experiments.

### Isolation and culture of NRVMs (neonatal rat ventricular myocytes)

NRVMs were isolated and maintained in culture as described previously [8], and transferred to maintenance medium [DMEM (Dulbecco's modified Eagle's medium)/M199 4:1 (v/v), 100 i.u./ml penicillin and streptomycin] 24 h after isolation. For RNAi experiments, the maintenance medium used did not contain antibiotics.

### Expression of recombinant FHL proteins

Plasmids encoding GST–FHL1 and GST–FHL2 fusion proteins were transformed into *Escherichia coli* BL21 (DE3) pLysS cells (Invitrogen). Cells were grown at 37°C in LB medium supplemented with 100 μg/μl ampicillin and 100 μM ZnCl_2_. When *D*_600_ reached 0.6–0.8, cells were induced to express the GST-fused proteins for 3 h by adding IPTG to 1 mM. Cells were then centrifuged at 5000 ***g*** for 5 min (4°C) in a Sorvall RC5B centrifuge (GSA rotor), pellets were resuspended in PBS containing 1% (v/v) Triton X-100 and Complete™ protease inhibitors (Roche) and suspensions were then sonicated on ice (three times for 15 s, Sonics Materials Ultrasonic Processor). For the purification of soluble recombinant GST and GST–FHL1, the lysate was cleared by centrifugation at 12000 ***g*** for 30 min (4°C) (rotor SS34) and the protein was purified by passing the cleared lysate through a glutathione–Sepharose 4B prepacked column (GE Healthcare) according to the manufacturer's instructions. For the purification of insoluble recombinant GST–FHL2, the lysate was centrifuged at 20000 ***g*** for 30 min (4°C) in a Beckman L60 ultracentrifuge (rotor SW 41 Ti). The pellet was then resuspended in PBS containing 1% (v/v) Triton X-100 and protease inhibitors and centrifuged again, as described above. The pellet was then dissolved in PBS containing 1% (v/v) Triton X-100, protease inhibitors and 8 M urea and centrifuged at 20000 ***g*** for 15 min (4°C). The supernatant, which contained the denatured recombinant GST–FHL2, was dialysed in refolding buffer containing 50 mM Tris/HCl, pH 8.0, 50 mM NaCl, 1 mM DTT and 0.1 mM ZnCl_2_. GST–FHL2 was purified using a glutathione–Sepharose 4B prepacked column, as noted above.

### GST pull-down assay

For the GST pull-down assay, lysates from ARVMs (treated with vehicle or 100 nM PMA for 10 min) or NRVMs (treated with vehicle or 10 nM ET1 for 10 min) prepared in lysis buffer [containing 50 mM Tris/HCl, pH 7.4, 0.05 mM ZnCl_2_, 2 mM DTT and 1% (v/v) Triton X-100] were clarified by centrifugation (25000 ***g***, 20 min, 4°C) in an Eppendorf 5417R centrifuge. Each supernatant was pre-cleared by incubation with glutathione–Sepharose 4B beads (GE Healthcare) for 1 h under rotation (4°C), then split in equal volumes and incubated for 2 h under rotation (4°C) with 5 μg of recombinant proteins (GST–FHL1, GST–FHL2 and GST) pre-bound for 1 h to glutathione–Sepharose 4B beads (4°C). The complexes were pelleted by centrifugation (1000 ***g***, 1 min, 4°C), washed three times in lysis buffer, resuspended in 2× SDS/PAGE sample buffer [125 mM Tris/HCl, pH 6.8, 4% (w/v) SDS, 20% (v/v) glycerol, 6% (v/v) 2-mercaptoethanol and 0.02% Bromophenol Blue], boiled and stored at −20°C until use.

### Immunoprecipitation

One day after isolation, NRVMs were transduced with adenoviral vectors expressing either GFP–PKD1 (at MOI of 50 pfu/cell) or GFP (at MOI of 10 pfu/cell). After 24 h, NRVMs were treated for 20 min with either ET1 (10 nM) or vehicle and GFP–PKD1 or GFP was immunoprecipitated by using the GFP-Trap®_A kit (Chromotek), according to the manufacturer's instructions. Briefly, NRVMs were lysed in a buffer containing 10 mM Tris, pH 7.5, 150 mM NaCl and 0.5 mM EDTA, supplemented with 0.5% Nonidet P40 and protease inhibitors. Lysates were incubated on ice for 30 min and subsequently centrifuged for 10 min at 20000 ***g*** (4°C). The cleared lysates were then diluted to a final volume of 1 ml with dilution buffer (containing 10 mM Tris/HCl, pH 7.5, 150 mM NaCl, 0.5 mM EDTA and supplemented with protease inhibitors) and incubated under rotation for 2 h (4°C) with GFP-Trap®_A beads. Beads were then washed three times in dilution buffer, resuspended in 100 μl of 2× SDS/PAGE sample buffer, boiled and stored at −20°C until use.

### *In vitro* phosphorylation assay

The *in vitro* phosphorylation assay was carried out as described previously [7]. Recombinant GST, GST–FHL1, GST–FHL2 and cTnI, alone or in combination (200 pmol each) as indicated, were incubated for 30 min at 37°C, in the absence or presence of active PKD (35 ng) together with 100 μM ATP, supplemented with [γ-^32^P]ATP in kinase assay buffer (30 mM Tris/HCl, pH 7.4, 15 mM MgCl_2_, 1 mM DTT and 0.05 mM ZnCl_2_). Proteins were resolved on 12% acrylamide gels, which were subsequently dried and subjected to autoradiography.

### RNAi

Synthetic siRNA sequences (denoted as ‘active’ siRNAs) and their respective scrambled counterparts (denoted as ‘scrambled’ siRNAs) with 3′ dTdT overhangs were purchased from Life Technologies. The sequences used were: 5′-GCCUGAAGUGCU-UUGACAA-3′ (active siRNA sequence for FHL1), 5′-GCCA-AUGCGUUGUAUGCAA-3′ (scrambled siRNA sequence for FHL1), 5′-GCAAGGACUUGUCCUACAA-3′ (active siRNA sequence for FHL2) and 5′-GCAUCAGCUGUAUCGACAA-3′ (scrambled siRNA sequence for FHL2). NRVMs were transfected with 40 nmol of active or scrambled siRNA sequences using Lipofectamine RNAimax (Life Technologies), 24 h after they were transferred to maintenance medium. The cells were then incubated for 48 h in transfection medium containing 22% (v/v) M199, 4% (v/v) horse serum and 74% (v/v) modified Dulbecco's balanced salt solution buffer (116 mM NaCl, 1 mM NaH_2_PO_4_, 0.8 mM MgSO_4_, 5.5 mM glucose, 32.1 mM NaHCO_3_ and 1.8 mM CaCl_2_, pH 7.2), after which time they were serum-starved for 3 h before treatment for 20 min with vehicle, ET1 (10 nM) or PE (3 μM, in the presence of 1 μM atenolol added 10 min before PE). Atenolol was used to inhibit any β-adrenergic receptor activation, which is known to counteract PKD activation [20,21].

### Pharmacological inhibition of PKD activity

In the experiments where the selective PKD inhibitor BPKDi was used, NRVMs were processed as described above for the RNAi experiments, but without the transfection step. BPKDi (3 μM) or vehicle was added 30 min before treatment with vehicle, ET1 or PE.

### Luciferase assays

NRVMs were transfected initially with active or scrambled siRNA sequences, as described above, and 24 h later with a combination of 0.6 μg of MEF2-*luc2P* luciferase reporter construct and 0.4 μg of *Renilla* luciferase reporter construct, the latter using Escort III transfection reagent (Sigma). The medium was changed to transfection medium without serum 5 h later and the cells incubated for a further 3 h, before they were treated with vehicle, ET1 (10 nM) or PE (3 μM, in the presence of 1 μM atenolol) for 18–24 h. Luciferase assays were performed using the Dual-Glo® Luciferase assay system and a GloMax® 20/20 luminometer (Promega), according to the manufacturer's instructions. In experiments where BPKDi was used, NRVMs were processed in a similar manner, but without the siRNA transfection step; BPKDi (3 μM) or vehicle was added 30 min before treatment with vehicle, ET1 or PE. Firefly luciferase activity was normalized against *Renilla* luciferase activity to account for transfection efficiency. Data were expressed relative to the scrambled siRNA-control group (in experiments where FHL protein levels were knocked down by RNAi) or to the vehicle-control group (in experiments where PKD activity was pharmacologically inhibited with BPKDi).

### Immunoblot analysis

Immunoblot analysis was performed as described previously [7], using specific antibodies for total or phosphorylated proteins as indicated. Specific protein bands were detected by ECL (GE Healthcare) and phosphorylation status was quantified using a calibrated densitometer (GS-800™, Bio-Rad Laboratories). Data were normalized to total protein loading (measured by densitometry in the respective immunoblots after Coomassie Blue staining) and expressed relative to an internal standard sample included in a separate lane, to permit consolidation of experiments analysed on different gels.

### Statistical analysis

Statistical comparisons were performed by one-way ANOVA followed by the Newman–Keuls post-hoc test. Quantitative data are given as means±S.E.M. and *P*<0.05 was considered significant.

## RESULTS

### PKD interacts with both FHL1 and FHL2

**Figure 1:**
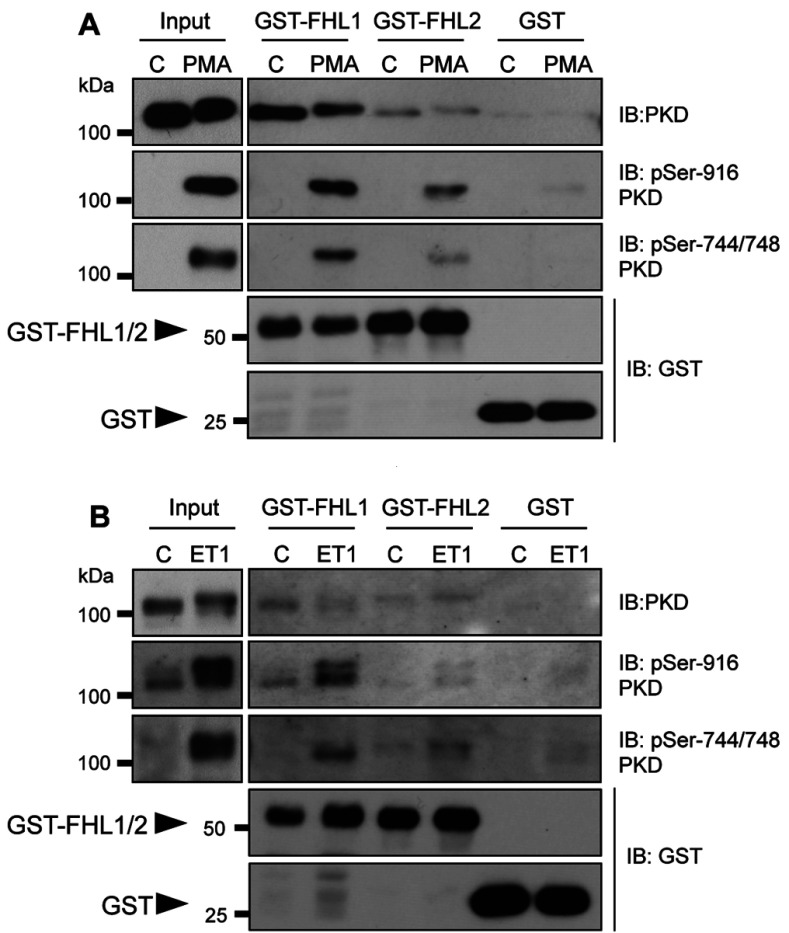
Pull-down of heterologously expressed or endogenous PKD1 with recombinant FHL1 and FHL2 (**A**) ARVMs transduced with AdV:wtPKD1 were treated with vehicle (C) or PMA (100 nM) for 10 min. Lysates were incubated with purified GST–FHL1, GST–FHL2 or GST bait proteins bound to glutathione–Sepharose beads and the PKD pull-down was assessed by immunoblot (IB) analysis using total or phospho-specific (pSer^916^ or pSer^744^/Ser^748^) PKD antibodies. Samples of the lysates before the pull-down assay (input, 10%) were also included in the immunoblot analyses and are shown in separate panels reflecting different exposure times. Bait protein content of the pull-downs was determined by immunoblot analysis using a GST antibody. (**B**) As in (**A**), but using lysates from NRVMs treated with vehicle (C) or ET1 (10 nM) for 10 min. Immunoblots are representative of three independent experiments.

The potential interactions of PKD with FHL1 and FHL2 were initially investigated through pull-down assays using recombinant GST–FHL1 and GST–FHL2 proteins and cell lysates from ARVMs that heterologously expressed mouse PKD1 following adenoviral gene transfer. ARVMs were treated with vehicle or PMA (to activate PKD) to ascertain the potential impact of PKD activation status on any interaction with FHL1 or FHL2. PKD1 protein was pulled down by both GST–FHL1 and GST–FHL2, but not by GST alone (Figure 1A, first panel), indicating a specific interaction with both FHL1 and FHL2. As expected, PMA markedly increased the phosphorylation status of PKD1 at Ser^744^/Ser^748^ and Ser^916^, reflecting activation of the kinase. Phosphorylated PKD1 associated with both FHL1 and FHL2 in this pull-down assay (Figure 1A, second and third panels), indicating that the PKD1–FHL1/2 association occurs independently of the PKD1 activation status. Bait protein (GST–FHL1, GST–FHL2 or GST) content was comparable in control and stimulated groups (Figure 1A, fourth and fifth panels).

To explore the interaction of endogenous PKD with FHL1 and FHL2, we additionally performed GST pull-down assays using cell lysates from NRVMs, in which PKD1 is more abundantly expressed than in ARVMs [3]. PKD1 was pulled down by GST–FHL1 and GST–FHL2, but not by GST alone, reflecting an interaction between the endogenous kinase and both FHL1 and FHL2 (Figure 1B, first panel). These interactions were also retained following the activation of endogenous PKD (Figure 1B, second and third panels), this time achieved by exposure to a physiological stimulus, ET1. As above, bait protein (GST–FHL1, GST–FHL2 or GST) content was comparable in control and stimulated groups (Figure 1B, fourth and fifth panels).

**Figure 2:**
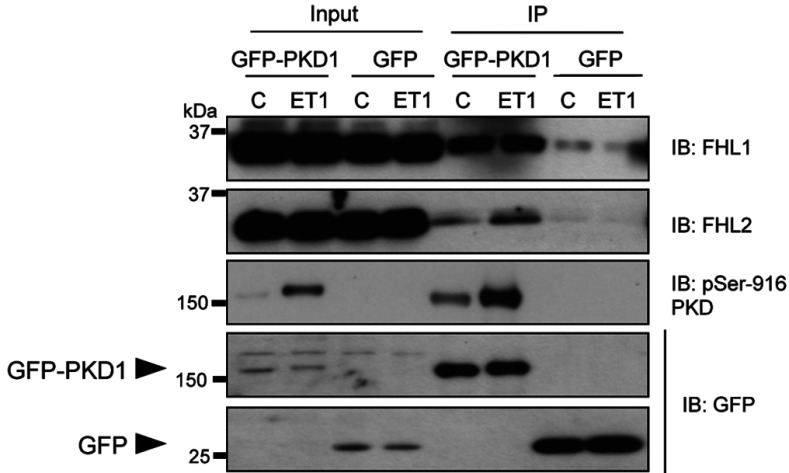
Co-immunoprecipitation of endogenous FHL1 and FHL2 with heterologously expressed PKD1 NRVMs transduced with AdV:GFP–PKD1 or AdV:GFP were treated with vehicle (C) or ET1 (10 nM) for 20 min and protein complexes associated with GFP–PKD1 or GFP were immunoprecipitated using GFP-Trap methodology. Association of GFP–PKD1 or GFP with endogenously expressed FHL1 and FHL2 was detected by immunoblot (IB) analysis of the immunoprecipitates (IP) using specific antibodies against FHL1 or FHL2. Expression and immunoprecipitation of GFP–PKD1 and GFP were verified by immunoblot analysis using a GFP antibody, whereas activation status of the PKD1 component of GFP–PKD1 was determined by immunoblot analysis using a phospho-specific pSer^916^ PKD antibody. Samples of the lysates before the IP (input, 10%) were also included in the immunoblot analyses. Data are representative of three independent experiments.

We next investigated whether the PKD1–FHL1/2 interaction occurs in the cellular environment of cardiac myocytes. To address this, we expressed GFP–PKD1 or GFP in NRVMs by adenoviral gene transfer and employed GFP-Trap methodology to efficiently immunoprecipitate protein complexes associated with these proteins. Immunoblot analysis revealed that endogenous FHL1 and FHL2 both co-immunoprecipitated with GFP–PKD1, but not with GFP, under basal conditions and after stimulation with ET1 (Figure 2, first and second panels). Activation of the PKD1 component of the heterologously expressed GFP–PKD1 fusion protein in response to ET1 was verified by immunoblot assessment of PKD autophosphorylation status at Ser^916^ (Figure 2, third panel). Immunoblot analysis with an anti-GFP antibody confirmed comparable expression and equally efficient immunoprecipitation of GFP–PKD1 and GFP in the different study groups (Figure 2, fourth and fifth panels). Taken together, the above results provide complementary biochemical evidence that FHL1 and FHL2 are novel interaction partners for PKD1 and that this interaction occurs independently of the activation status of the kinase.

### FHL1 and FHL2 are not phosphorylated by PKD

**Figure 3:**
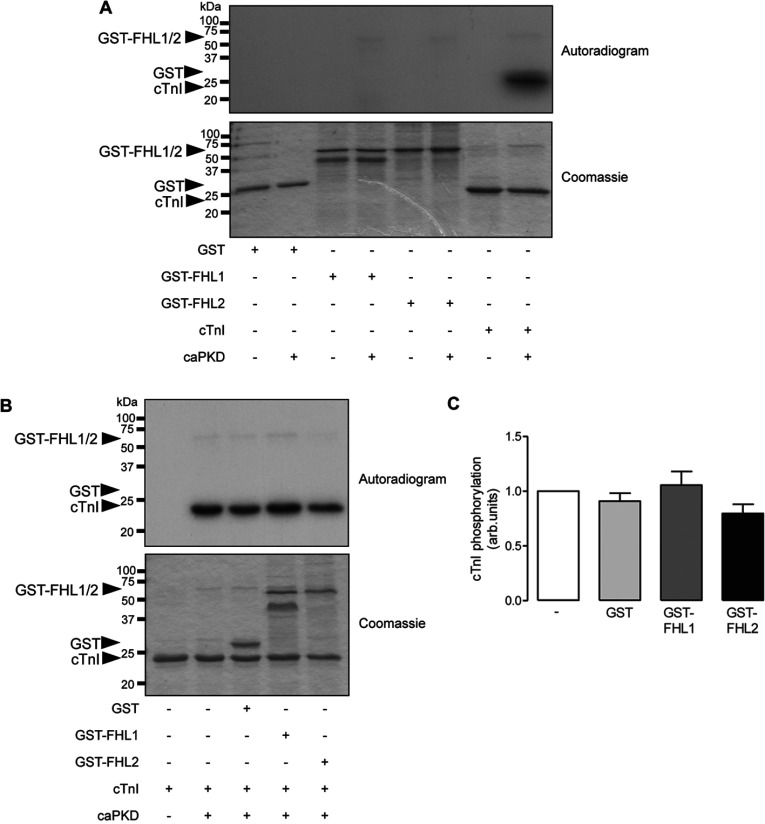
FHL1 and FHL2 are not PKD substrates (**A**) Autoradiograms of *in vitro* phosphorylation assays using recombinant caPKD and recombinant GST, GST–FHL1, GST–FHL2 or cTnI protein as substrate. (**B**) Assays were also conducted using cTnI as substrate, in the absence or presence of additional recombinant GST, GST–FHL1 or GST–FHL2 protein. (**C**) Histogram showing quantitative data from experiments as illustrated in (**B**). Reactions were performed in the presence of [γ-^32^P]ATP and proteins were resolved by SDS/PAGE (12% gel). Protein loading was confirmed by Coomassie Blue staining. Data are representative of three independent experiments, and the quantitative data in (**C**) are means±S.E.M. (*n*=3).

Our previous studies that built on the discovery of novel cardiac PKD1 interaction partners through a yeast two-hybrid screen [7] have led to the identification of cTnI and cMyBP-C as PKD substrates and the mapping and functional analysis of the pertinent substrate phosphorylation sites [7–9]. In an analogous approach, we explored whether FHL1 and FHL2 are also PKD substrates, by performing IVK (*in vitro* kinase) assays in which the caPKD (constitutively active catalytic domain of PKD) was incubated with recombinant GST, GST–FHL1 or GST–FHL2 proteins in the presence of [γ-^32^P]ATP. Autoradiographic analysis revealed no detectable phosphate incorporation into GST, GST–FHL1 or GST–FHL-2, even though robust phosphorylation of cTnI was readily detectable (Figure 3A). To explore whether FHL1 or FHL2 might affect directly the catalytic activity of PKD, we also performed IVK reactions in which caPKD was incubated with cTnI in the absence or presence of each of the GST-fused proteins. These experiments revealed that cTnI phosphorylation was not affected by the presence of GST, GST–FHL1 or GST–FHL2 (Figure 3B). Such findings indicate that FHL1 and FHL2 are not PKD substrates, which is consistent with the fact that the amino acid sequences of these proteins do not contain PKD substrate consensus phosphorylation motifs, as revealed by *in silico* analysis (http://phospho.elm.eu.org). They also suggest that interaction with FHL1 or FHL2 does not inhibit the catalytic activity of caPKD towards an established substrate, cTnI.

### FHL1 and FHL2 facilitate PKD phosphorylation in cardiac myocytes

**Figure 4:**
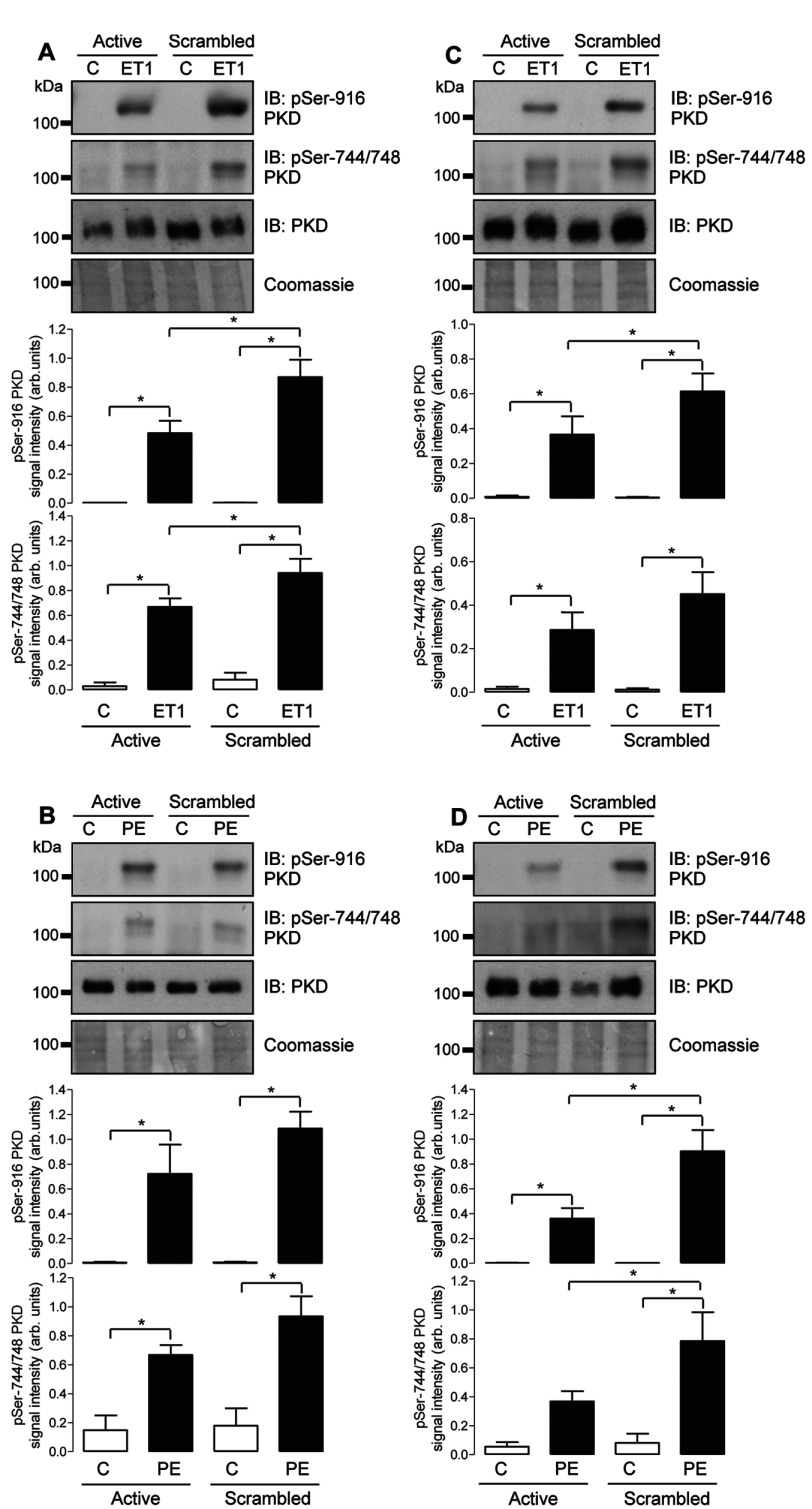
Effect of FHL1 or FHL2 knockdown on ET1- and PE-induced phosphorylation of endogenous PKD NRVMs were transfected with either scrambled siRNA or active siRNA duplexes targeted at FHL1 (**A** and **B**) or FHL2 (**C** and **D**) transcripts. After 48 h, cells were treated with vehicle (C) or ET1 (10 nM) (**A** and **C**) or vehicle (C) or PE (3 μ M) (**B** and **D**) for 20 min. Phosphorylation status of endogenous PKD at Ser^916^ and Ser^744^/Ser^748^ was determined by immunoblot (IB) analysis using appropriate phospho-specific antibodies. Protein loading was confirmed using an anti-PKD antibody and by Coomassie Blue staining. Individual immunoblots illustrate representative experiments, and histograms show quantitative data as means±S.E.M. (*n*=7–8). *P<0.05.

To explore the potential roles of FHL1 and FHL2 in regulating the activation and functions of endogenous PKD in cardiac myocytes, we applied a loss-of-function approach and knocked down FHL1 or FHL2 protein expression by RNAi through transfection of NRVMs with synthetic siRNA duplexes specific for each FHL mRNA sequence. Transfection with FHL1 siRNA caused an approximately 75% decrease in FHL1 protein expression relative to control cells transfected with a scrambled siRNA sequence, with no apparent effect on FHL2 expression (Supplementary Figures S1A and S1B at http://www.biochemj.org/bj/457/bj4570451add.htm). Similarly, transfection with FHL2 siRNA caused a comparable decrease in FHL2 protein expression relative to cells transfected with a scrambled siRNA sequence, with no compensatory change in FHL1 expression (Supplementary Figures S1C and S1D). We then examined the consequences of the selective knockdown of each FHL isoform on PKD activation in response to stimulation with ET1 or PE. As illustrated in Figure 4, both stimuli significantly increased PKD autophosphorylation at Ser^916^ and transphosphorylation at Ser^744^/Ser^748^ in control cells transfected with scrambled siRNA. Knocking down FHL1 expression significantly attenuated the increase in PKD phosphorylation in response to ET1 (Figure 4A), but had no significant effect on the increase in PKD phosphorylation in response to PE (Figure 4B). In contrast, knockdown of FHL2 expression significantly attenuated the increases in PKD phosphorylation in response to both ET1 (Figure 4C) and PE (Figure 4D), with a greater inhibitory effect on the latter. These findings suggest that FHL1 and FHL2 facilitate PKD activation by multiple neurohormonal stimuli, potentially in a stimulus-dependent manner, by promoting transphosphorylation of the PKD activation loop. Interestingly, the effects of simultaneous knockdown of both FHL1 and FHL2 on PKD phosphorylation were similar to the effects of selective FHL2 knockdown, with no indication of an additive effect (Supplementary Figure S2 at http://www.biochemj.org/bj/457/bj4570451add.htm).

FHL2 has been reported to promote the stability of myocardin and myocardin-related transcription factor-A by protecting these proteins from proteasome-mediated degradation [22], raising the possibility that, if FHL1 or FHL2 similarly protected PKD protein from degradation, the apparent effects of FHL knockdown on PKD phosphorylation described above might actually reflect a decrease in total PKD protein levels. We therefore analysed total PKD protein levels in samples after FHL1 or FHL2 knockdown. Despite a tendency, there was no significant reduction in PKD protein expression with knockdown of either FHL1 or FHL2 (Supplementary Figure S3 at http://www.biochemj.org/bj/457/bj4570451add.htm). Thus the negative effects of reduced FHL isoform expression by RNAi on ET1- and PE-induced increases in PKD autophosphorylation at Ser^916^ and transphosphorylation at Ser^744^/Ser^748^ (Figure 4) most likely indicate an attenuation of PKD activation, rather than simply reflecting reduced PKD protein expression.

### FHL1 and FHL2 facilitate HDAC5 phosphorylation in cardiac myocytes

**Figure 5:**
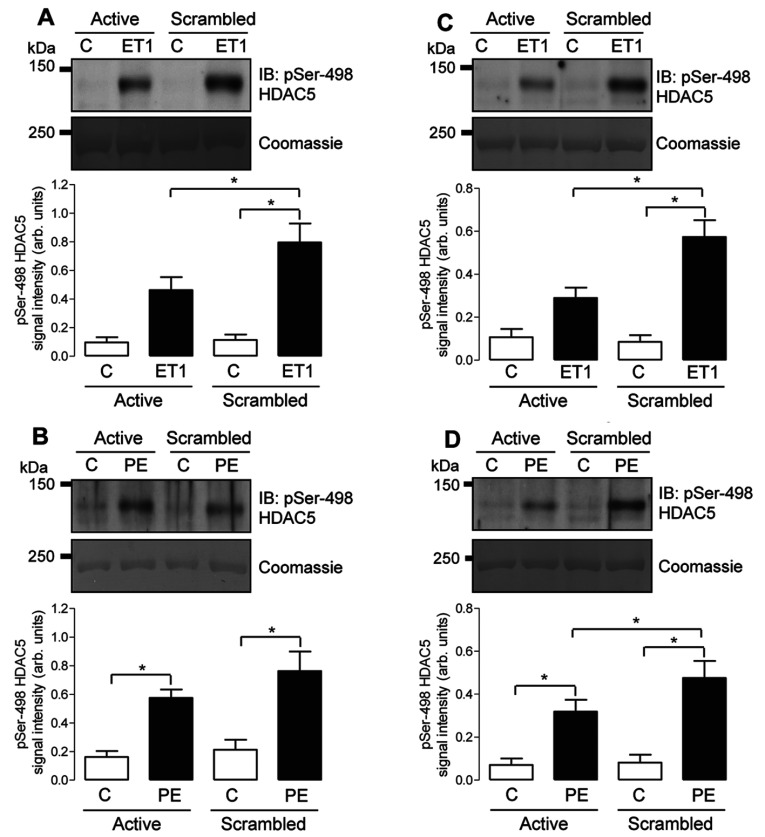
Effect of FHL1 or FHL2 knockdown on ET1- and PE-induced phosphorylation of endogenous HDAC5 at Ser^498^ NRVMs were transfected with either scrambled siRNA or active siRNA duplexes targeted at FHL1 (**A** and **B**) or FHL2 (**C** and **D**) transcripts. After 48 h, cells were treated with vehicle (C) or ET1 (10 nM) (**A** and **C**) or vehicle (C) or PE (3 μM) (**B** and **D**) for 20 min. The phosphorylation status of endogenous HDAC5 was determined by immunoblot (IB) analysis using a phospho-specific pSer^498^ HDAC5 antibody. Protein loading was confirmed by Coomassie Blue staining. Individual immunoblots illustrate representative experiments, and histograms show quantitative data as means±S.E.M. (*n*=7–8). *P<0.05.

To explore whether the attenuation of PKD activation is paralleled by reduced phosphorylation of a functionally important cellular PKD substrate, we also studied the effects of FHL1 and FHL2 knockdown on the phosphorylation status of HDAC5 at Ser^498^, an established PKD substrate in cardiac myocytes [10,11]. Selective FHL isoform knockdown in NRVMs attenuated HDAC5 phosphorylation in response to stimulation with ET1 or PE (Figure 5), in a manner that mirrored the effects of these loss-of-function interventions on PKD phosphorylation (Figure 4). Thus selective knockdown of FHL1 expression significantly attenuated the increase in HDAC5 phosphorylation in response to ET1 compared with control cells transfected with a scrambled siRNA sequence (Figure 5A), but had a non-significant effect on the increase in HDAC5 phosphorylation in response to PE (Figure 5B). In contrast, selective knockdown of FHL2 expression significantly attenuated the increases in HDAC5 phosphorylation in response to both ET1 (Figure 5C) and PE (Figure 5D). Therefore it appears that, in response to neurohormonal stimulation in NRVMs, FHL1 and FHL2 facilitate not only PKD activation, but also phosphorylation of the downstream PKD substrate HDAC5. Once again, the effects of simultaneous knockdown of both FHL1 and FHL2 on HDAC5 phosphorylation were similar to the effects of selective FHL2 knockdown, with no indication of an additive effect (Supplementary Figure S2).

### FHL1 and FHL2 do not regulate MEF2 activation in cardiac myocytes

**Figure 6:**
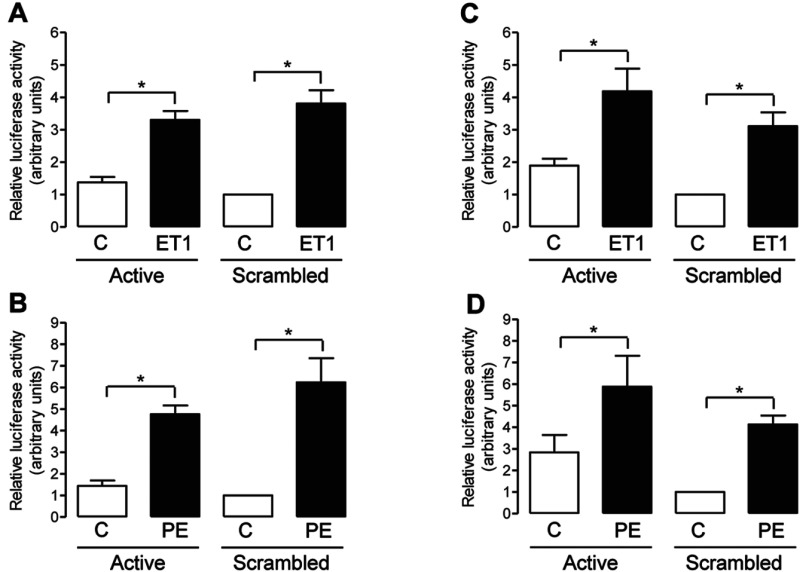
Effect of FHL1 or FHL2 knockdown on ET1- and PE-induced activation of endogenous MEF2 NRVMs were transfected with either scrambled siRNA or active siRNA duplexes targeted at FHL1 (**A** and **B**) or FHL2 (**C** and **D**) transcripts. After 24 h, cells were co-transfected with a 3×MEF2-firefly luciferase reporter vector and a *Renilla* luciferase control vector and treated with vehicle (C) or ET1 (10 nM) (**A** and **C**) or vehicle (C) or PE (3 μM) (**B** and **D**) for a further 18–24 h. Luciferase activity in cell lysates was assessed by *in vitro* luminescence assays, with luciferase activity normalized for *Renilla* luciferase activity in each sample to correct for transfection efficiency. Histograms show quantitative data as means±S.E.M. (*n*=4). *P<0.05.

PKD-mediated phosphorylation of HDAC5 at Ser^498^ is believed to trigger HDAC5 nuclear export, with the consequent de-repression of MEF2 activity [13,14]. We therefore hypothesized that knocking down FHL1 or FHL2 expression might also have an impact on ET1- and PE-induced MEF2 activation, as a consequence of attenuated PKD activation and reduced HDAC5 phosphorylation. To test this hypothesis, MEF2 transcriptional activity was monitored in NRVMs that were transfected with a MEF2-luciferase reporter. As expected, stimulation of control NRVMs transfected with scrambled siRNA with either ET1 or PE significantly increased MEF2 activity (Figure 6). Surprisingly, however, transfection with active siRNA targeted at FHL1 (Figures 6A and 6B) or FHL2 (Figures 6C and 6D) transcripts had no significant effect on ET1- and PE-induced increases in MEF2 activity, despite again achieving marked reductions in FHL protein expression (Supplementary Figure S4 at http://www.biochemj.org/bj/457/bj4570451add.htm).

**Figure 7:**
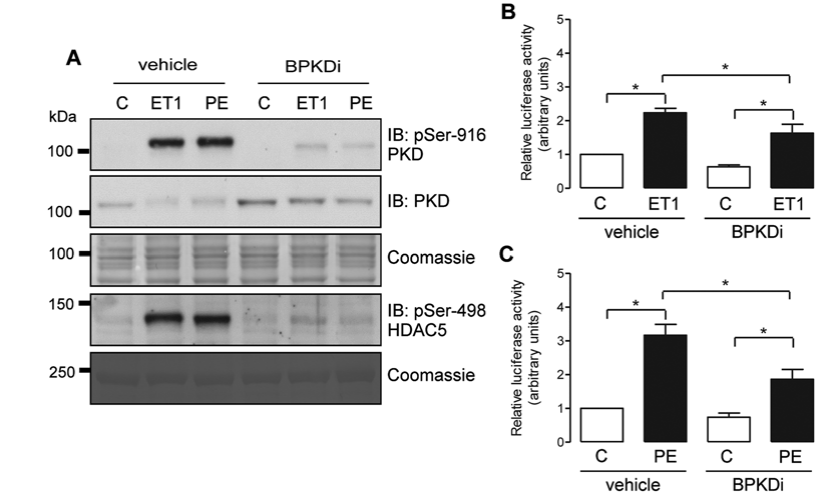
Effect of pharmacological PKD inhibition on ET1- and PE-induced HDAC5 phosphorylation and MEF2 activation (**A**) NRVMs were treated with vehicle (C), ET1 (10 nM) or PE (3 μM) for 20 min, following a 30-min pre-treatment with vehicle or the BPKDi (3 μM). PKD autophosphorylation at Ser^916^, total PKD expression and HDAC5 phosphorylation at Ser^498^ were assessed by immunoblot (IB) analysis using appropriate antibodies, as indicated. Membranes were stained with Coomassie Blue to confirm equal protein loading. Data are representative of three independent experiments. (**B** and **C**) NRVMs were co-transfected with a 3×MEF2-firefly luciferase reporter vector and a *Renilla* luciferase control vector and treated with vehicle (C), ET1 (10 nM) or PE (3 μM) for 18–24 h, following a 30-min pre-treatment with vehicle or the BPKDi (3 μM). Luciferase activity in cell lysates was assessed by *in vitro* luminescence assays, with luciferase activity normalized for *Renilla* luciferase activity in each sample to correct for transfection efficiency. Histograms show quantitative data as means±S.E.M. (*n*=4). *P<0.05.

One possible explanation for this lack of effect of FHL isoform knockdown on MEF2 activation, despite a significant attenuation of PKD activation, is that under our experimental conditions PKD activity is not an important mediator of MEF2 activation in response to ET1 or PE. To help confirm the role of PKD activity in MEF2 activation in NRVMs, therefore, we also investigated the effects of the selective PKD inhibitor BPKDi [20,23] under the same experimental conditions. Consistent with previous work [23], pre-treatment of NRVMs with BPKDi completely abolished ET1- and PE-induced increases in both PKD autophosphorylation and HDAC5 phosphorylation (Figure 7A). Furthermore, pre-treatment with BPKDi significantly attenuated ET1- and PE-induced increases in MEF2 activity (Figures 7B and 7C). Thus it appears that FHL1 and FHL2 facilitate PKD activation and HDAC5 phosphorylation, but not MEF2 activation following ET1 and PE stimulation, despite the fact that PKD activity is indeed an important mediator of both HDAC5 phosphorylation and MEF2 activation under these experimental conditions.

## DISCUSSION

The present study provides novel information concerning the regulation of PKD activity and functions in cardiac myocytes, through the following principal findings: (i) PKD interacts with two members of the FHL protein family, FHL1 and FHL2; (ii) FHL1 and FHL2 are not PKD substrates and do not inhibit PKD catalytic activity; (iii) FHL1 and FHL2 differentially regulate PKD activation after neurohormonal stimulation; and (iv) FHL1 and FHL2 do not regulate MEF2 activity after neurohormonal stimulation.

FHL1 and FHL2 are non-enzymatic proteins that are expressed primarily in striated muscles, including the heart [18,24,25]. Their importance for normal muscle function is evidenced by the fact that mutations in the genes encoding these proteins *fhl1* and *fhl2*, are associated with various skeletal and cardiac myopathies [25]. The multiple LIM domains comprising FHL1 and FHL2 form a tandem zinc-finger structure that provides a modular protein-binding interface, through which FHL1 and FHL2 function as adaptors or scaffolds to support the assembly of multimeric protein complexes and regulate the localization and activity of their partners [24,25]. Examples include specific interactions between FHL1 and FHL2 with an array of transcription factors, such as AP-1 (activator protein 1) [26] and HIF-1 (hypoxia-inducible factor 1) [27]. FHL proteins may co-activate or co-repress these transcription factors, depending on the identity of the interaction partner, the stimulus and the cell type [24–27], thereby exerting regulatory effects on gene expression. In addition, FHL1 and FHL2 have been reported to form biologically significant interactions with the α5β1 and α7β1 integrins [28,29], the muscle-specific RING finger proteins MuRF1 and MuRF2 [30], the metabolic enzymes creatine kinase, adenylate kinase and phosphofructokinase [19] and other protein kinases like ERK2 (extracellular-signal-regulated kinase 2) [31,32]. Such interactions define the functional roles of FHL1 and FHL2 in regulating biological processes such as cell division, differentiation, migration and metabolism [24,25]. The present study adds PKD to the growing list of interaction partners for FHL1 and FHL2 and provides evidence that these interactions are of functional importance in regulating PKD activity in cardiac myocytes in response to neurohormonal stimulation.

Previous studies have explored the roles that FHL1 and FHL2 may play in cardiac myocyte (patho)physiology through the targeted disruption of *fhl1* and *fhl2* in mice. Such studies have revealed that the global loss of FHL1 or FHL2 protein does not impair cardiovascular development, or cause any spontaneous cardiac phenotype [32–34]. They have also shown that the targeted deletion of either *fhl1* or *fhl2* does not induce a compensatory change in the cardiac expression of the other isoform [32,34], which is reminiscent of our observations with individual knockdown of FHL1 or FHL2 expression in NRVMs (Supplementary Figure S1). Nevertheless, when the hearts of gene-targeted mice were subjected to different types of stress stimuli, some overt differences in the manifestation of pathological phenotypes became apparent. For example, in comparison with their wild-type littermates, *fhl1*^−/−^ mice have been reported to develop diminished cardiac hypertrophy following TAC (transverse aortic constriction) or cardiac-specific Gα_q_ overexpression, suggesting a pro-hypertrophic role for FHL1 [32]. On the contrary, *fhl2*^−/−^ mice presented with a comparable magnitude of cardiac hypertrophy relative to wild-type controls after TAC [34,35], but mounted an exaggerated hypertrophic response after chronic β-adrenergic receptor stimulation by isoproterenol infusion [33], suggesting an anti-hypertrophic role for FHL2 in the latter setting. Thus the roles that FHL1 and FHL2 play in regulating cardiac hypertrophy in the *in vivo* setting may be isoform-specific and dependent on the nature of the stress stimulus that triggers the hypertrophic response. Interestingly, at least in some settings, FHL1 and FHL2 appear to have differential effects on cardiac myocyte ERK activity [31,32], raising the possibility that the ERK pathway may be critical in mediating the regulation of cardiac hypertrophy by FHL proteins. Furthermore, recent evidence suggests a role for FHL2 in the regulation of calcineurin/nuclear factor of activated T-cell signalling, another pro-hypertrophic pathway [35]. FHL2 represses calcineurin, with which it co-localizes at the sarcomere [35]. Loss of FHL2 from the sarcomere has been observed in human heart failure samples, suggesting that changes in the subcellular distribution of the protein may be associated with disease development [36].

In our *in vitro* studies in NRVMs, selective knockdown of either FHL1 or FHL2 inhibited PKD activation by pro-hypertrophic neurohormonal stimuli and attenuated the downstream phosphorylation of HDAC5, albeit with some differences in their impact on the responses to ET1 against PE (discussed later). Nevertheless, MEF2 activation by these stimuli was unaffected by FHL1 or FHL2 knockdown (Figure 6). These data suggest that FHL protein-mediated regulation of PKD activity is unlikely to play a critical role in controlling MEF2-driven cardiac myocyte transcriptional reprogramming towards hypertrophy, despite its impact on HDAC5 phosphorylation. In this regard, although pharmacological PKD inhibition abolished the neurohormonal induction of PKD autophosphorylation and HDAC5 transphosphorylation, it incompletely (albeit significantly) inhibited MEF2 activation in our studies (Figure 7). It is possible, therefore, that a combination of PKD-independent mechanisms, such as the phosphorylation of class II HDACs by CaMKII [37,38] or GRK5 (G-protein-coupled receptor kinase 5) [39], or their redox-mediated nuclear export [20,40], or indeed HDAC-independent mechanisms mediated by p38-MAPK (mitogen-activated protein kinase) [41], ERK5 [42,43] or the acetyltransferase p300 [44], may be sufficient to induce significant MEF2 activation and thereby MEF2-driven transcriptional reprogramming towards cardiac hypertrophy. In this context, it is also important to note that pharmacological inhibition of PKD activity has been shown not to attenuate cardiac hypertrophy in different models in the rat *in vivo* [45], although cardiac-specific deletion of PKD1 in the mouse significantly inhibited cardiac hypertrophy in response to TAC or chronic isoproterenol infusion [15]. A plausible interpretation of such data is that PKD1 regulates cardiac hypertrophy through a mechanism that is independent of its kinase activity, in which case FHL1- and FHL2-mediated regulation of neurohormonal PKD activation would be more likely to regulate other PKD-mediated functions. In the cardiac context, PKD activity has been implicated in the regulation of myofilament calcium sensitivity and dynamics [7–9], voltage-gated calcium channel activity [46], protection against ischaemia/reperfusion injury [47] and insulin resistance [48], and the potential roles of FHL proteins in regulating such processes warrant further investigation.

The possible mechanism(s) through which FHL1 and FHL2 may regulate PKD activation also require consideration. Previous studies in rat cardiac myocytes have shown that neurohormonal PKD activation is achieved through the phosphorylation of its activation loop, principally by the novel PKC isoform PKCε [5,21]. Interestingly, proteomic analysis of the cardiac PKCε signalling complex has identified FHL2 as a component protein [49]. Moreover, PKCε association with another LIM-domain protein, enigma homologue, has been proposed to play a key role in its substrate targeting [50]. Given that ET1- and PE-induced phosphorylation of PKD at Ser^744^/Ser^748^ was diminished by FHL1 or FHL2 knockdown in the present study (Figure 4), it is plausible that FHL proteins may act as scaffolds that facilitate the co-localization of PKCε and PKD and thus the phosphorylation and activation of the latter, in a signal-responsive manner. Additionally, the differential effects of FHL1 and FHL2 knockdown on ET1- compared with PE-induced PKD phosphorylation at Ser^744^/Ser^748^ and Ser^916^ (Figure 4) suggest that the two FHL proteins might associate with distinct subcellular pools of PKD downstream of the ET1 and α_1_-adrenergic receptors. In this context, it is interesting to note recent evidence which suggests that ET1 and PE may induce spatiotemporally distinct patterns of PKD activation in cardiac myocytes [4]. The potential contribution of FHL isoforms to such compartmentalized regulation also requires further investigation.

In conclusion, in the present study, we identify FHL1 and FHL2 as novel interaction partners for PKD in cardiac myocytes and we show that FHL1 and FHL2 regulate PKD activation in response to neurohormonal stimulation. Although such regulation affects HDAC5 phosphorylation, it does not control neurohormonal MEF2 activation, suggesting that PKD regulation by FHL proteins is likely to be of functional importance in PKD-mediated (patho)physiological processes other than MEF2-driven transcriptional reprogramming towards cardiac hypertrophy.

## Online data

Supplementary data
