# Supplementary material for: Four-and-a-half LIM domains proteins are novel regulators of the protein kinase D pathway in cardiac myocytes
Source: Biochem J. 2014 Jan 10;457(Pt 3):451–61. doi: 10.1042/BJ20131026 (PMC3927927; doi:10.1042/BJ20131026)
Supplement: Supplementary data [file bj4570451add.pdf]

BJ Signal

Biochem. J. (2014) **457**, 451–461 (Printed in Great Britain) doi:10.1042/BJ20131026

online data

SUPPLEMENTARY ONLINE DATA

Four-and-a-half LIM domains proteins are novel regulators of the protein kinase D pathway in cardiac myocytes

Konstantina STATHOPOULOU*†, Friederike CUELLO*†, Alexandra J. CANDASAMY*, Elizabeth M. KEMP*, Elisabeth EHLER*, Robert S. HAWORTH* and Metin AVKIRAN*[1]

*Cardiovascular Division, King's College London British Heart Foundation Centre, London SE1 7EH, U.K.
†Institute of Experimental Pharmacology and Toxicology, University Medical Center Hamburg-Eppendorf, Martinistr. 52, Hamburg 20146, Germany

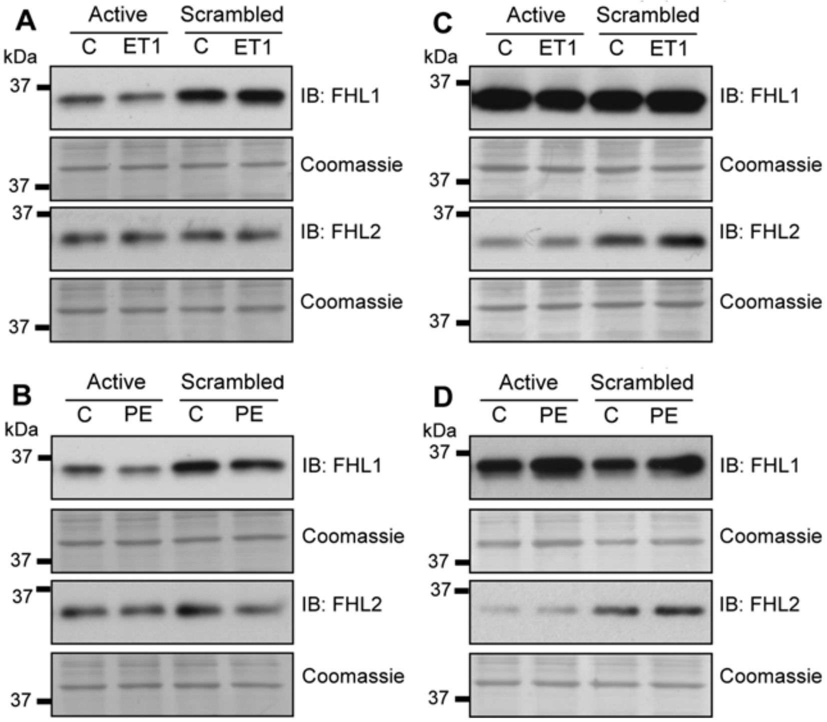

Figure S1 Efficiency and selectivity of FHL1 or FHL2 knockdown by siRNA transfection

NRVMs were transfected with either scrambled siRNA or active siRNA duplexes targeted at FHL1 (**A** and **B**) or FHL2 (**C** and **D**) transcripts. After 48 h, cells were treated with vehicle (C), ET1 (10 nM) or PE (3 μM) for 20 min. FHL1 and FHL2 protein expression levels were assessed by immunoblot (IB) analysis using selective antibodies as indicated. Protein loading was confirmed by Coomassie Blue staining. Molecular masses are indicated in kDa on the left.

[1] To whom correspondence should be addressed (email metin.avkiran@kcl.ac.uk).

© 2014 The Author(s)
The author(s) has paid for this article to be freely available under the terms of the Creative Commons Attribution Licence (CC-BY) (http://creativecommons.org/licenses/by/3.0/) which permits unrestricted use, distribution and reproduction in any medium, provided the original work is properly cited.

Biochemical Journal www.biochemj.org

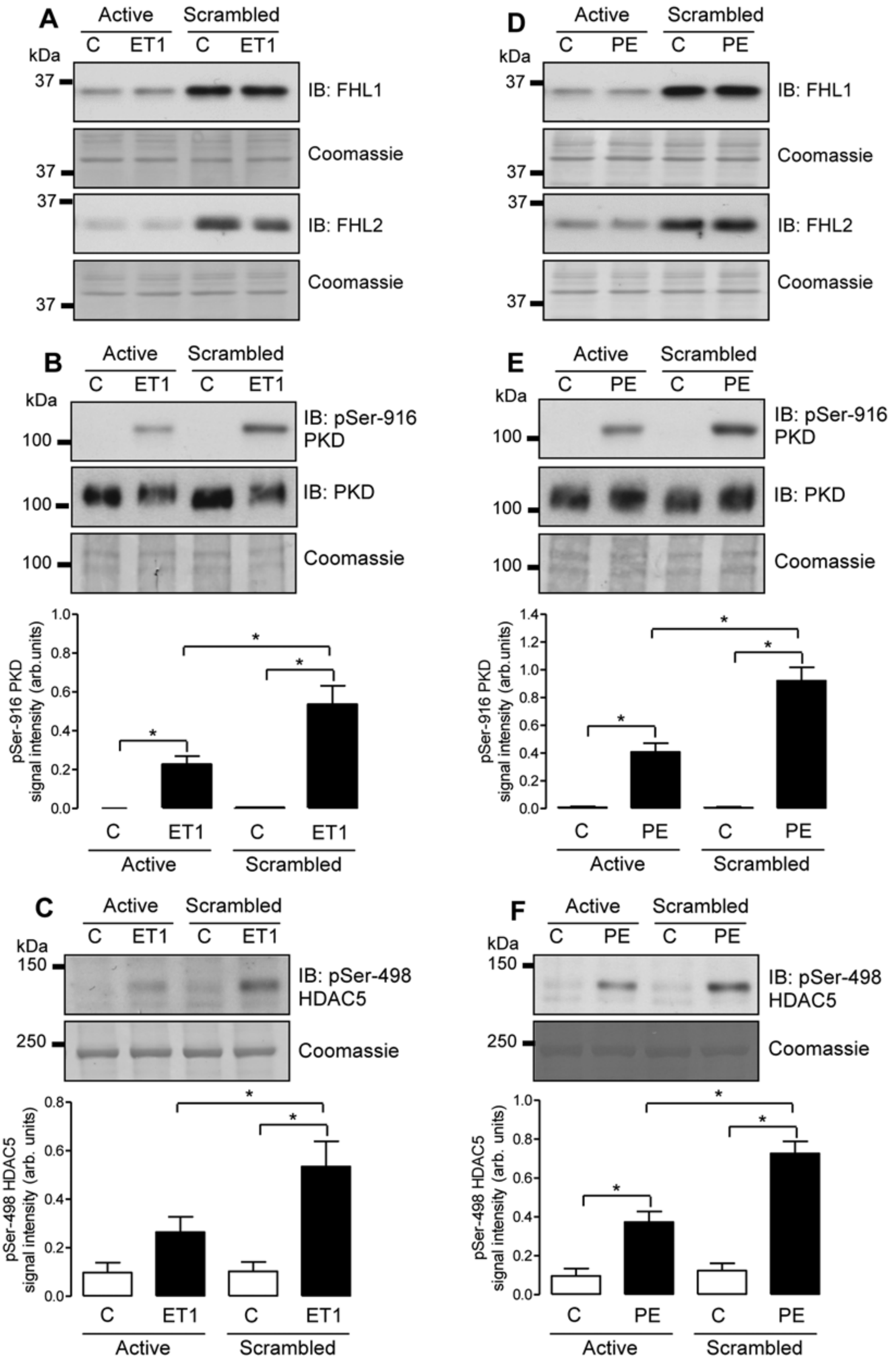

Figure S2 Effect of simultaneous FHL1 or FHL2 knockdown on ET1- and PE-induced phosphorylation of endogenous PKD and HDAC5

NRVMs were concomitantly transfected with either scrambled siRNA or active siRNA duplexes targeted at FHL1 and FHL2 transcripts. After 48 h, cells were treated with vehicle (C) or ET1 (10 nM) (**A**–**C**), or vehicle (C) or PE (3 μM) (**D**–**F**) for 20 min. FHL1 and FHL2 protein expression levels were assessed by immunoblot (IB) analysis using selective antibodies as indicated (**A** and **B**). Phosphorylation status of endogenous PKD (**B** and **E**) and HDAC5 (**C** and **F**) was determined by immunoblot (IB) analysis using a phospho-specific $pSer^{916}$ PKD and $pSer^{498}$ HDAC5 antibodies respectively. Protein loading was confirmed by an anti-PKD antibody and Coomassie Blue staining. Individual immunoblots illustrate representative experiments, and histograms show quantitative data as means$\pm$S.E.M. ($n = 8$). $^{*}P < 0.05$. Molecular masses are indicated in kDa on the left.

© 2014 The Author(s)

The author(s) has paid for this article to be freely available under the terms of the Creative Commons Attribution Licence (CC-BY) (http://creativecommons.org/licenses/by/3.0/) which permits unrestricted use, distribution and reproduction in any medium, provided the original work is properly cited.

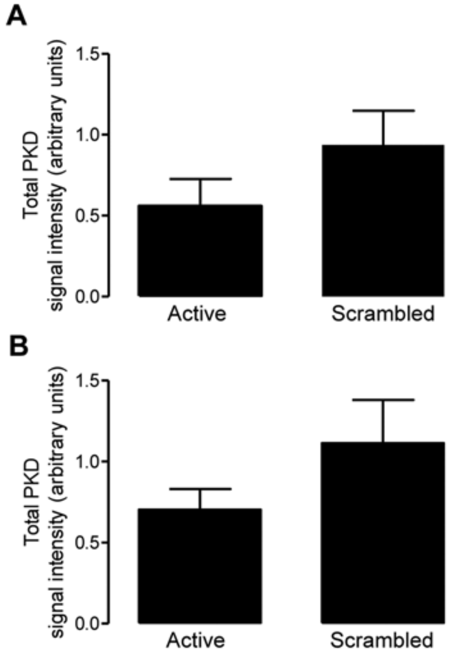

Figure S3 Effect of FHL1 or FHL2 knockdown on PKD protein expression

NRVMs were transfected with either scrambled siRNA or active siRNA duplexes targeted at FHL1 (**A**) or FHL2 (**B**) transcripts. After 48 h, PKD protein expression was detected by immunoblot analysis using a selective antibody. Histograms show quantitative data as means±S.E.M. ($n = 8$).

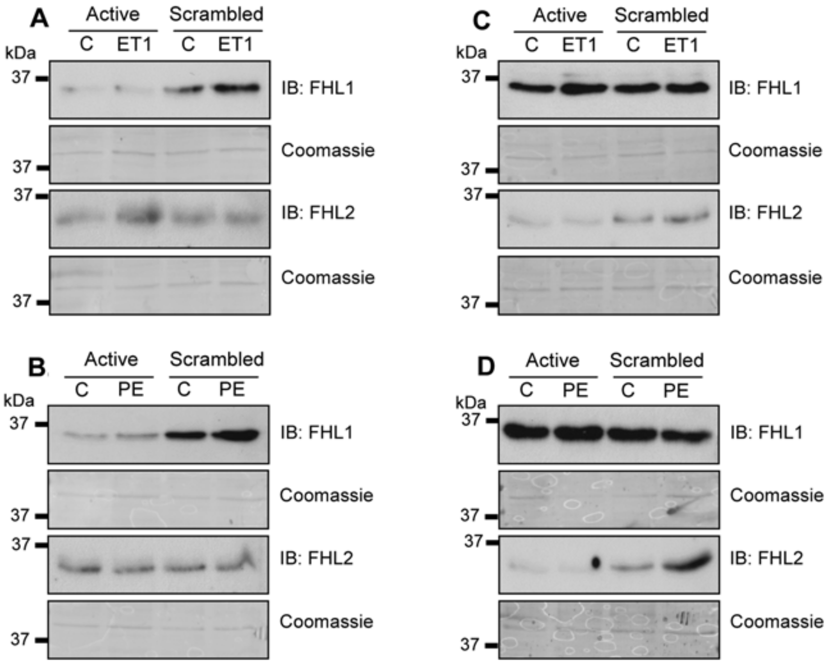

Figure S4 Efficiency and selectivity of FHL1 or FHL2 knockdown by siRNA transfection in cardiac myocytes additionally transfected with luciferase reporters

NRVMs were transfected with either scrambled siRNA or active siRNA duplexes targeted at FHL1 (**A** and **B**) or FHL2 (**C** and **D**) transcripts. After 24 h, cells were co-transfected with a 3×MEF2-firefly luciferase reporter vector and a *Renilla* luciferase control vector and treated with vehicle (C), ET1 (10 nM) or PE (3 μM) for a further 18–24 h. FHL1 and FHL2 protein expression levels were assessed by immunoblot (IB) analysis using selective antibodies as indicated. Protein loading was confirmed by Coomassie Blue staining. Molecular masses are indicated in kDa on the left.

Received 1 August 2013/31 October 2013; accepted 13 November 2013
Published as BJ Immediate Publication 13 November 2013, doi:10.1042/BJ20131026

© 2014 The Author(s)
The author(s) has paid for this article to be freely available under the terms of the Creative Commons Attribution Licence (CC-BY) (http://creativecommons.org/licenses/by/3.0/) which permits unrestricted use, distribution and reproduction in any medium, provided the original work is properly cited.
